# “I need to learn how to cite without the website”: using think alouds to understand students’ struggles with academic referencing

**DOI:** 10.1042/ETLS20253027

**Published:** 2025-12-12

**Authors:** Jayne L. Dennis

**Affiliations:** 1 School of Biological and Behavioural Sciences, Queen Mary University of London, London, U.K

**Keywords:** citation, referencing, student experience, think aloud, transition to university

## Abstract

Many students struggle with the mechanical process of academic referencing when they transition into higher education. Previous research has typically utilised questionnaires and/or interviews to gain retrospective insight into students’ cognitions while referencing. The think-aloud method, which asks participants to verbalise their thoughts while performing a task, was utilised while 30 undergraduate participants completed a referencing exercise, followed by semi-structured interviews. Thematic analysis of transcripts identified that students could articulate why referencing was important, although they struggled with the mechanical process of referencing, often experiencing nervousness and a dislike of referencing. As a generation of information consumers, participants held little regard for the nuance of academic referencing, instead placing importance on finding sources via search engines using minimal information such as first author and article title. The referencing exercise used in this study received positive feedback and could be incorporated into taught sessions to better support students’ development of this core academic skill.

## Introduction

Students often perceive the transition into higher education as a challenging time [1], caused by many changes in their personal and academic lives [2]. One challenge students face is assimilation into the community of academic practice [3], which includes academic writing and referencing [4]. For many students, referencing is a skill they need to acquire as part of the transition into higher education [5].

Academic referencing encompasses both the practice of using ideas from sources [6] and the mechanical process of in-text citation and compiling a reference list or bibliography at the end of the work [4]. Although the mechanical process might be ‘not difficult in itself’ [4: p352], students often struggle with academic referencing [7], giving rise to multiple errors in their work [8].

Previous research has typically utilised questionnaires and/or interviews to gain retrospective insight into students’ cognitions while referencing [5,7,9,10]. However, Eccles and Arsal [11] argued that participants’ reports of their cognitive processes may be of limited validity and better insight can be gained through a ‘think-aloud’ method. As the name suggests, the think-aloud method asks participants to verbalise their thoughts while performing a task. This provides an insight into at least a subset of thoughts involved in the task and complements insights gained through retrospective approaches such as interviews [11]. Think alouds have been used in a variety of contexts, such as survey design, text comprehension and second language learning [12].

This action research aimed to enhance understanding of students’ struggles with academic referencing by using think alouds while completing a referencing exercise alongside semi-structured interviews.

## Methods

All first-year undergraduate students enrolled on a biological science (i.e. biochemistry, biology, zoology, biomedical sciences, neuroscience, medical genetics or pharmacology) or law programme in September 2021 in one London Russell Group institution were invited by email to participate. Rather than aiming to compare responses between disciplines, the aim was to gain insight from more than one group of students.

Respondents completed an online survey that collected demographic information. Survey respondents were then invited by email to participate in the think-aloud referencing exercise held in person.

**Figure 1:**
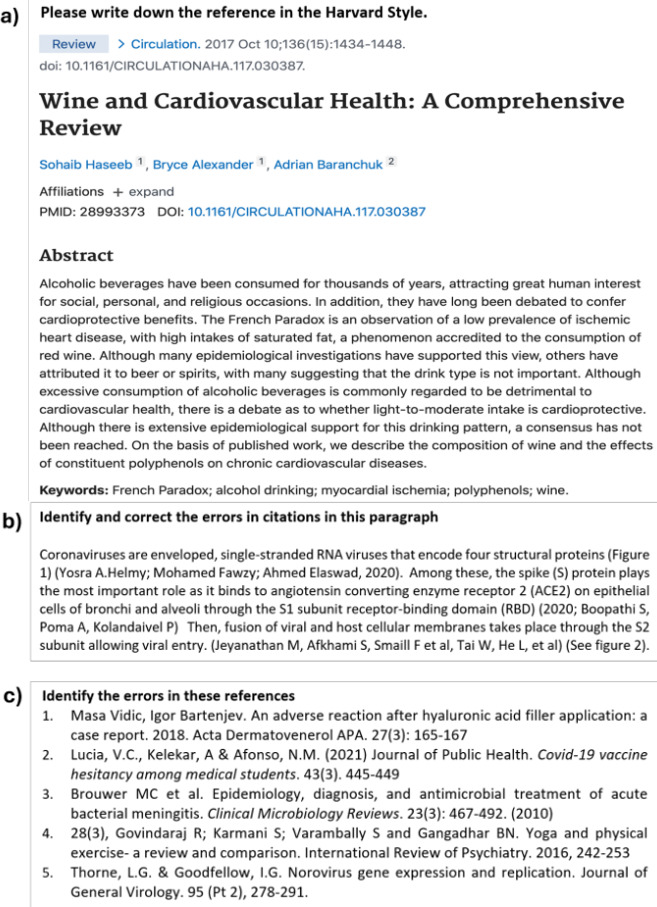
Examples of exercises participants completed in the think-aloud activity. (**A**) Participants were required to extract relevant information from the image to construct the reference as it should be written in the reference list. (**B**) Participants were required to identify and correct citation errors in the paragraph. (**C**) Participants were required to identify and correct the errors in the reference list.

The in-person research began by obtaining written informed consent, and participants practised thinking aloud while completing a logic problem. Participants studying a biological science then chose to complete a referencing exercise using either the Harvard or Vancouver style; law students completed an exercise based on the OSCOLA style, which is commonly used by the discipline. All activities (Figure 1) commenced with approximately five images of sources, for example, from PubMed, and participants were tasked with writing the full reference for each source, as it should appear in a reference list. Participants were then presented with a reference list of ten sources; they had to identify formatting errors, such as a misplaced year or omitted journal name. Finally, one paragraph contained in-text citations, some of which were incorrect and had to be corrected. A semi-structured interview followed; this explored study habits and opinions on referencing. The in-person activities were conducted by final year undergraduate student researchers as part of their capstone project; they received training on research methods from their academic supervisor. The in-person sessions took no more than one hour each to complete. Sessions were audio-recorded, manually transcribed and checked for accuracy before conducting thematic analysis.

Approximately 900 students were invited to participate; 102 completed the survey, and 30 subsequently completed the in-person activities, drawn equally from biological sciences and law programmes (Table 1). Three student researchers conducted activities with participants acting individually, six participants per student researcher. Another student researcher conducted activities with four groups of three participants to gain insight into the influence of peers while referencing.

**Table 1:** Participant demographic information.

Participant ID	Programme	Referencing style	Gender	Participation
D1	Law	OSCOLA	Female	Individual, SR1
D2	Medical genetics	Harvard	Female	Individual, SR1
D3	Law	OSCOLA	Female	Individual, SR1
D4	Neuroscience	Harvard	Female	Individual, SR1
D5	Biomedical sciences	Vancouver	Male	Individual, SR1
D6	Medical genetics	Harvard	Male	Individual, SR1
E1	Law	OSCOLA	Female	Individual, SR2
E2	Law	OSCOLA	Female	Individual, SR2
E3	Law	OSCOLA	Female	Individual, SR2
E4	Biomedical sciences	Harvard	Female	Individual, SR2
E5	Biomedical sciences	Harvard	Female	Individual, SR2
E6	Law	OSCOLA	Female	Individual, SR2
F1	Biology	Harvard	Female	Individual, SR3
F2	Law	OSCOLA	Male	Individual, SR3
F3	Law	OSCOLA	Female	Individual, SR3
F4	Law	OSCOLA	Female	Individual, SR3
F5	Medical genetics	Vancouver	Female	Individual, SR3
F6	Biomedical sciences	Harvard	Female	Individual, SR3
E1-1	Law	OSCOLA	Female	Group 1, SR4
E1-2	Law	OSCOLA	Male	Group 1, SR4
E1-3	Law	OSCOLA	Female	Group 1, SR4
E2-1	Law	OSCOLA	Female	Group 2, SR4
E2-2	Law	OSCOLA	Female	Group 2, SR4
E2-3	Law	OSCOLA	Female	Group 2, SR4
E3-1	Neuroscience	Harvard	Male	Group 3, SR4
E3-2	Neuroscience	Harvard	Male	Group 3, SR4
E3-3	Biomedical sciences	Harvard	Female	Group 3, SR4
E4-1	Biomedical sciences	Harvard	Female	Group 4, SR4
E4-2	Biomedical sciences	Harvard	Female	Group 4, SR4
E4-3	Biomedical sciences	Harvard	Female	Group 4, SR4

OSCOLA is the referencing style commonly used within the law discipline.

SR, student researcher.

Thematic analysis [13] began with transcript familiarisation. An inductive approach identified provisional codes based on prevalence within the data; theme rationalisation followed. Quotations below were taken from participants studying biological sciences, although all reported themes were equally evident among law participants.

Ethical concerns were identified using a standard framework [14], and the study was approved by the local Ethics of Research Committee, approval number QMERC20.493. To incentivise participation, participants completing the in-person research could opt in to a draw for one of two £50 vouchers for an online retailer.

## Results and discussion

Thematic analysis identified that students believed referencing was an important academic practice. However, referencing elicited negative emotions because students struggled to identify and correctly format relevant information in reference lists. The think-aloud exercises revealed these struggles arose from poor instruction and a reliance on websites which generate reference information. Instead, students preferred to focus on information necessary for finding sources online, such as the first author’s name and the article title. The student participants in this study valued the referencing activities as an instructional method for learning the mechanical process of referencing.

### Importance of referencing

Participants believed that referencing was important. When importance was rated on a scale from 1 to 10, where 10 is the most important, 20 participants gave an average rating of 9.4; the range was 7–10, with one student rating importance as ‘11’, for emphasis. A further three participants made qualitative comments on the importance of referencing, and six did not directly answer the question.

When asked why referencing was important, a common response was a desire to avoid being accused of plagiarism, as reported by others previously [5]. Nonetheless, when participants were asked whether, in the absence of plagiarism penalties, they still thought referencing was important, participants stressed the importance of evidencing sources and proper attribution of authorship. Participants also believed that including references gave weight to their arguments and enabled staff to verify the claims and information:

“It’s like I'm practically stealing another person’s work if don't really credit them at the end” (E4-1)

“Your tutor needs to, or whoever (is) reading it, needs to be able to find your citation or reference in order to fact check it”. (F6)

Many participants also associated the act of referencing with professionalism:

“It’s a behaviour in the scientific community as well, so it’s kind of like shaking someone’s hand at the start of a business meeting. If you don't reference, you're not doing right. It’s like etiquette almost”. (D4)

Similar rationale for including references in academic work has been reported previously [8,15,16].

### Emotive act

Referencing was an emotive act, as reported previously [3,5,8]. While completing the exercise, participants expressed nervousness and a dislike of referencing – despite having volunteered to contribute to a research study on referencing.

“I do not like referencing not gonna lie. … And, like, every time I do it, I get, like, so scared. … This actually gives me, like, (post-traumatic stress disorder) to, like, (International Baccalaureate) biology” (E4-1)

“I hate referencing. … Referencing is very annoying” (E4-2)

At interview, when asked about how they felt when referencing, a few participants declared no specific emotions, although most expressed negative emotions such as stress, nervousness or boredom:

“I'm kind of stressed sometimes and I was like, if I get it wrong, it feels like stealing, so, like I want to get it right and not feel like an idiot” (D2)

“I'm very nervous about referencing because I've never really done it before” (E5)

“Bored! It’s quite nit-picky!” (D4)

### Struggles and referencing

Students’ ‘struggles’ with academic referencing have been reported previously [5]. During the think-aloud referencing exercise, participants tended to struggle with two aspects of referencing: the ability to identify the relevant information from the images in order to write the reference and correct formatting of the reference:

“This is a journal or some sort. That’s probably a textbook. Maybe. It might be. It might be a journal. Oh, it’s a book” (E4-3)

“Referencing, at first, it blew my mind away because I was looking at all of these names and numbers and I just got so confused”. (F5)

These struggles may be attributed to students using online tools to complete referencing tasks for them:

“Oh, I use websites, you know the converter that does it for you. … Since I use website, I couldn't remember the order. … I need to learn how to cite without the website” (F1)

Previous work suggested that a lack of referencing training resulted in students using automatic referencing websites [5]; the think-aloud method revealed that using such websites may contribute to a negative feedback loop whereby students never learn the referencing skill, thereby perpetuating anxiety around referencing.

As with other research, participants named a variety of resources to assist with referencing their coursework assignments, including Cite This For Me, Google Scholar, MyBib, YouTube videos and university resources [8].

“Vancouver, because that’s the stuff I've watched on YouTube before and they recommend that if you do, like, a science related degree, Vancouver is typically the referencing guide that the university would recommend”. (F5)

Several participants also spoke about using the referencing function within Microsoft Word, unlike students interviewed by Howard and Tummon [8]. The difference is attributable to the inclusion of Microsoft Word in the referencing session attended by the present participants.

### Grading referencing

After completing the referencing task and reviewing the model answers, participants were asked to assign a grade to their performance in the task. The suggested range was one to ten, with ten being high quality work. 24 participants gave a quantitative response; the average was 4.7 (range 1–8), reflecting the common presence of errors in their work. When justifying their grade, participants acknowledged the effort invested in the task but, ultimately, their work lacked accuracy:

“We did do our best, but we definitely had quite a few mistakes” (E4-1)

When Howard and Tummon [8] reviewed submitted summative assessments, they also reported extensive errors in citations and references across disciplines and referencing styles.

Our participants were subsequently asked if they should be marked down in their summative assignments for errors in their referencing technique. A couple of participants acknowledged the need for accurate referencing, consistent with professional academic practice [17]:

“I think you should get marked down for it, because if you're trying to give out like a quality essay, then all of it needs to be of the same quality”. (D4)

Nonetheless, participants generally thought it was sufficient to provide the minimal information that enables the reader to locate the source. Typically, this comprised the first author’s name, the article title and/or a web address to the article. Participants generally attached less importance to details such as the journal and volume number and reference formatting.

“I think you can just do author and title” (E4)

“I’d probably just put on the URL” (D5)

“if it’s completely different to what the lecturer asked, then, yeah, I think there should be some sort of penalty. But if it’s not too different and there’s, like, one or two elements missing from the actual reference, then I don't think it should require marking down”. (E3-2)

These findings are consistent with previous research which identified a tension between students’ intuitive internet use while performing online learning tasks and their difficulties with the structured approaches required for formal academic learning [18].

### Instruction

Participants were asked about any instruction received in academic referencing. Like previous research [10], a small minority of participants commented that referencing was required at school. At university, most participants were aware that referencing was addressed in a taught session in the first weeks at university. These sessions were typically brief and didactic, emphasising the importance of referencing with little practical information on how to reference.

“I don't think it was clear enough. They explained to us the importance of referencing, but not exactly how to do it”. (E4)

Participants said they would have liked the training session to be more practical and interactive. Previous studies have also identified students’ desire for clear guidelines, supported by interactive workshops [5] included as part of the curriculum [6,8].

Several participants commented positively on the activities completed as part of this research, with further positive feedback from students who worked in small groups.

“I think giving us some exercises to do will help us to understand it better”. (F1)

“this kind of exercise made me kind of think about what, what is actually in the reference, rather than just referencing and then moving on to the next one” (E3-2)

“other people could spot my mistakes and we could discuss together what the referencing was like and, like, whether we're not supposed to reference it like this or like that” (E4-3)

### Significance and implications for practice

This study echoes previous findings about students’ opinions on the importance of referencing, even though students struggle with the mechanical process and they experience negative emotions while referencing. The think-aloud exercises in this study provided valuable insights into why these struggles exist: online searches and reliance on websites which generate reference lists appear to inhibit students’ understanding of the relevance of bibliographic information such as volume number. Instead, students preferred to generate reference lists which included, for example, the first author’s name, the article title and/or weblinks.

These findings do not imply that students should be prohibited from using tools, websites or artificial intelligence (AI) when referencing. Instead, the findings suggest that students value practical and interactive activities, especially when conducted in small groups, which improve their understanding of the academic referencing process. Although some participants would have preferred starting with the mistake correction activity, research suggests that learners who first struggle to recall a correct answer and are then provided with clarification learn more than those who simply read the correct answer [19]. Consequently, it is recommended that the exercises are utilised in the same order as presented here, i.e. generate a reference list using images from a reference database such as PubMed and then correct errors in citations and a reference list.

When deploying such activities, they could be used to not only increase students’ understanding of the mechanical process of referencing but also to enable students to critically appraise the output from referencing and AI tools and websites. This will empower students to identify and correct errors in both received output and in the work they submit for summative assessment. In turn, such activities have the potential to ameliorate students’ struggles and negative emotions while referencing and assimilating into the wider community of academic practice.

### Limitations

Although larger than most previous qualitative studies, the limited sample size is susceptible to bias through incomplete representation of the broader student population. Participants, although drawn from two disparate disciplines, were enrolled at one institution; this limits the generalisability of results. Except for the degree programme, transcripts were not analysed with respect to demographic variables that may have informed participants’ previous experiences of academic referencing. Nonetheless, the congruence between many findings and previous research gives credence to the novel findings elicited through the think-aloud exercise.

## Conclusion

The think-aloud exercise and semi-structured interviews revealed that students found academic referencing to be a challenging and emotive activity, despite acknowledging the importance of correctly attributing work. As a generation of information consumers, today’s undergraduate students seem to hold little regard for the traditions of academic writing and referencing, and instead they place importance on being able to find sources via search engines using minimal information such as first author and article title. These findings suggest that, if we continue using referencing conventions such as Harvard or Vancouver, students should learn the mechanical process of referencing through practical exercises similar to those used in this research study.

**Summary points**

- Undergraduate students often struggle with, and experience negative emotions while performing, the mechanical process of academic referencing.
- Thinking aloud while conducting a referencing exercise revealed that participants place importance on searching for articles using information such as title and author, over publishing conventions, such as volume and issue.
- Participants would like to be taught the mechanical process of referencing using activities such as the one completed in this study.

## Open Access

This article has been published open access under our Subscribe to Open programme, made possible through the support of our subscribing institutions, learn more here: https://portlandpress.com/pages/open_access_options_and_prices#conditional

## Abbreviations

AI: artificial intelligence
